# We should avoid the term “fluid overload”

**DOI:** 10.1186/s13054-018-2141-7

**Published:** 2018-09-11

**Authors:** Jean-Louis Vincent, Michael R. Pinsky

**Affiliations:** 1 Department of Intensive Care, Erasme University Hospital, Université Libre de Bruxelles, Route De Lennik 808, 1070 Brussels, Belgium; 2 Department of Critical Care Medicine, University of Pittsburgh, Pittsburgh, PA USA

**Keywords:** Hypervolemia, Blood volume, Edema, Fluid administration, Shock

Using the right word or phrase to describe a specific pathologic process/patient diagnosis and/or status is important, not only within the intensive care unit team, but also when we communicate with external consultants. This is not just a question of semantics. Using incorrect terms can lead to misunderstanding and even to incorrect therapeutic decisions. For example, it is not uncommon to see clinicians examining an edematous patient, saying that the patient has “fluid overload” or “hypervolemia” or both and proposing fluid restriction and/or diuretics as the logical strategy, when often during the acute phases of resuscitation from circulatory shock this approach may be inappropriate.

A fundamental determinant of cardiac output and its ability to vary in response to changing metabolic demands is the body’s effective circulating blood volume. Total circulating blood volume is distributed throughout the circulatory system into vessels that can initially be filled without changing their distending pressures because they merely alter their conformation rather than stretch, rather like a large empty balloon that will not have any measurable distending pressure when air is initially blown into it. Once filled beyond this volume, however, wherein conformational changes alone cannot accept more air without requiring the balloon’s walls to stretch, the distending pressure will start to increase. The volume in the balloon below this point is called its unstressed volume. In the body, this unstressed volume accounts for between 60 and 70% of the total circulating blood volume [1]. As illustrated in Fig. 1, the amount of unstressed volume can be rapidly and dynamically changed by changing blood flow distribution, increasing surrounding tissue pressure, or increasing venomotor tone [2]. The effective circulating blood volume reflects the proportion of blood volume above the unstressed volume and is essentially independent of arterial pressure, because arterial pressure is dissipated across the high resistance arterioles, analogous to a “vascular waterfall”. The stressed blood volume relative to the venous compliance defines the mean circulatory filling pressure, which is the back pressure to venous flow from the body back to the right ventricle. The primary reason for giving fluids during resuscitation is to increase the stressed circulatory blood volume, thus causing mean circulatory filling pressure to rise. If the heart can handle this increased driving pressure, then venous return increases causing cardiac output to rise. If the effective circulating blood volume is low, then mean circulatory filling pressure is also low, and if the effective circulating blood volume is high, then mean circulatory filling pressure is also high.

**Fig. 1:**
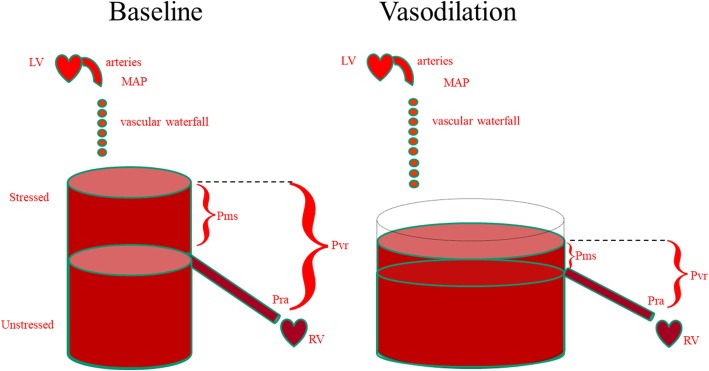
The relationship between unstressed and stressed blood volume and mean systemic pressure (*Pms*) and its independence from mean arterial pressure (*MAP*) and right atrial pressure (*Pra*). Pra is important because the driving pressure for venous return (*Pvr*) is the pressure difference between Pms and Pra. Thus, for the same blood volume, vasodilation, by increasing unstressed volume, decreases both Pms and Pvr, causing cardiac output to decrease. *LV* left ventricle, *RV* right ventricle

Accordingly, *hypo*volemia refers to a low intravascular blood volume, wherein mean systemic filling pressure is low and there is a net flux of water and electrolytes out of the interstitial and tissue spaces into the blood stream. This tends to support blood volume but with decreased tissue rigidity and loss of vascular reserve. At the opposite end of the spectrum, *hyper*volemia refers to an excessive blood volume, wherein mean systemic circulatory pressure is high, causing a net fluid loss into the interstitium resulting in some edema formation. The causes of hypervolemia can be complex and include renal failure, congestive heart failure, or liver failure, especially when these conditions are associated with overzealous fluid administration. Hypervolemia is also sometimes associated with hyponatremia, especially in patients with cirrhosis, renal failure, or heart failure, reflecting a defect in free water excretion. Unfortunately, hypervolemia is not a condition with which the body is prepared to cope, as all stress states are usually associated with hypovolemia. This is why clinicians often need to intervene in the management of hypervolemia with the use of diuretics or ultrafiltration. In essence, the body defends bleeding and *hypo*volemia. *Hyper*volemia is not a priority. Nevertheless, in the perioperative setting, both hypovolemia and hypervolemia are associated with several unfavorable outcomes, including acute renal failure, respiratory complications, and increased length of stay, costs, and even 30-day mortality [3, 4].

Although hypervolemia is always associated with some edema, the reverse is not always true, i.e., edema is not always associated with hypervolemia, particularly in acutely ill patients, especially those with sepsis or other types of intravascular inflammatory responses (e.g., pancreatitis, burns), who often have altered capillary permeability. In such patients, protein-rich fluid leaks from the intravascular to the interstitial space even when mean circulatory filling pressure is low, leading to combined hypovolemia and peripheral edema. Hypoalbuminemia, common in critically ill patients, can exacerbate such edema formation and slow its resolution. If vasoplegia coexists, then mean circulatory filling pressure can be even lower, even though absolute blood volume may not be as decreased as would be the case if tone were normal. This is the rationale for the proposed initial aggressive fluid bolus resuscitation step in the management of septic shock. Thus, in these patients one often sees functional or real hypovolemia associated with increased total body water and generalized edema. These patients often need more intravascular fluids if in shock, not fluid restriction or diuretics. However, whether aggressive fluid resuscitation or combined volume expansion and vasopressor infusion is the most efficacious initial therapy is unclear. Vasopressor infusions in vasoplegic states minimize the volume infusion requirements needed to achieve acceptable mean arterial pressure targets in volume responsive patients.

Even when the capillaries are intact, an abrupt increase in hydrostatic pressures, related, for example, to acute heart failure or an acute adrenergic discharge, can result in edema formation stemming from fluid extravasation from the intravascular compartment into the interstitium. More than 40 years ago, Da Luz and co-workers measured plasma volume under these conditions and showed that it is typically reduced [5]. Since then, a prudent fluid challenge has become part of the standard management of cardiogenic shock [6], because although hypervolemia is always associated with edema, edema can be associated with an increased, a normal, or a decreased blood volume and administering fluid can be harmful or beneficial depending on the underlying cause.

Based on this construction, it should be clear that fluid overload is a poorly-defined term, often confused with hypervolemia, but not synonymous. If, as is often the case, the term is used based solely on the presence of edema, errors in management may occur, with fluids being withheld or diuretics administered just because the edema is assumed to indicate the presence of excess fluid. It is important to remember that once patients become stable following initial management for acute circulatory insufficiency, diuretics should be administered to aid in fluid removal, but only when there is actual hypervolemia. For those who have needed resuscitation, this should be limited to the stabilization or the de-escalation phase [6].

In summary, the terms hypervolemia and fluid overload are often used interchangeably, yet they do not have the same meaning. “Fluid overload” may vaguely refer to excess total body water content associated with edema, but within medical circles it would be better if the term were avoided completely. The word “hypervolemia” is sufficient to indicate an excess in circulating blood volume and, if present, needs to be properly documented before a strategy of fluid restriction and/or diuretics is applied.

## Abbreviations

MAP: Mean arterial pressure
Pms: Mean systemic pressure
Pra: Right atrial pressure
Pvr: Driving pressure for venous return
RV: Right ventricle
